# Objective and subjective measures of prior sleep–wake behavior predict functional connectivity in the default mode network during NREM sleep

**DOI:** 10.1002/brb3.1172

**Published:** 2018-12-04

**Authors:** Rebecca S. Wilson, Stephen D. Mayhew, David T. Rollings, Aimee Goldstone, Joanne R. Hale, Andrew P. Bagshaw

**Affiliations:** ^1^ Centre for Human Brain Health University of Birmingham Birmingham UK; ^2^ School of Psychology University of Birmingham Birmingham UK; ^3^ Department of Neurophysiology Queen Elizabeth Hospital Birmingham UK; ^4^ Center for Health Sciences SRI International Menlo Park California; ^5^ Clinical Physics and Bioengineering University Hospital Coventry and Warwickshire Coventry UK

**Keywords:** default mode network, functional connectivity, functional MRI, habitual sleep behavior, sleep

## Abstract

**Introduction:**

Prior sleep behavior has been shown to correlate with waking resting‐state functional connectivity (FC) in the default mode network (DMN). However, the impact of sleep history on FC during sleep has not been investigated. The aim of this study was to establish whether there is an association between intersubject variability in habitual sleep behaviors and the strength of FC within the regions of the DMN during non‐rapid eye movement (NREM) sleep.

**Methods:**

Wrist actigraphy and sleep questionnaires were used as objective and subjective measures of habitual sleep behavior, and EEG‐functional MRI during NREM sleep was used to quantify sleep.

**Results:**

There was a significant, regionally specific association between the interindividual variability in objective (total sleep time on the night before scanning) and subjective (Insomnia Severity Index) measures of prior sleep–wake behavior and the strength of DMN FC during subsequent wakefulness and NREM sleep. In several cases, FC was related to sleep measures independently of sleep stage, suggesting that previous sleep history effects sleep FC globally across the stages.

**Conclusions:**

This work highlights the need to consider a subject's prior sleep history in studies utilizing FC analysis during wakefulness and sleep, and indicates the complexity of the impact of sleep on the brain both in the short and long term.

## 1 INTRODUCTION

The fundamental purpose of sleep remains an active topic of debate, although there is agreement that it is crucial for optimal functioning of the brain's cognitive domains, which are adversely affected by poor or insufficient sleep (Hobson, 2005). Cognitive functions arise from complex interactions between distributed brain regions, processes which can be investigated noninvasively in the human brain using functional MRI (fMRI). In particular, functional connectivity (FC) examines the temporal correlation between the activity of different brain regions and has proved to be an important tool to characterize brain function (Buckner, Krienen, & Yeo, 2013) During sleep, FC tends to reduce, indicating a loss of the regional interactions that support cognitive functions (Duyn, 2012; Picchioni, Duyn, & Horovitz, 2013). The default mode network (DMN), a set of regions which are relatively deactivated during active task performance (Fox, Zhang, Snyder, & Raichle, 2009), has been shown to have the most pronounced alterations to FC with sleep onset, with the most consistent observations being a reduction in FC between the frontal regions and those more posterior (Horovitz et al., 2008; Larson‐Prior et al., 2009, 2011; Sämann et al., 2011; Spoormaker, Gleiser, & Czisch, 2012; Uehara et al., 2014; Wilson et al., 2015).

In addition to changes in FC following sleep onset, past sleep behaviors including habitual sleep duration estimated from wrist actigraphy (Khalsa et al., 2016) and experimentally induced, acute sleep deprivation (De Havas, Parimal, Soon, & Chee, 2012; Gujar, Yoo, Hu, & Walker, 2010; Sämann et al., 2010; Yeo, Tandi, & Chee, 2015) have been linked with reductions in DMN FC during wakefulness. The variability of subjective measures of sleep across participants has also been shown to be associated with differences in their waking FC, including self‐reported total sleep time (TST) the previous night (Killgore, Schwab, & Weiner, 2012) and level of daytime sleepiness (Ward et al., 2013). In particular, daytime sleepiness has been associated with reduced FC in regions of the DMN (Ward et al., 2013) and the thalamus (Killgore et al., 2015).

To date, there has been no examination of the potential links between prior sleep history and the subsequent FC during sleep. The fact that prior sleep history is informative about waking FC, and the differences in sleep architecture that are observed between normal sleep and recovery sleep following sleep deprivation (Bonnet, 2005; Bonnet, Berry, & Arand, 1985; Borbély & Achermann, 1999; Brunner, Dijk, Tobler, & Borbély, 1990), leads us to hypothesize that differences in habitual sleep patterns across individuals will be reflected in differences in FC during sleep. As the DMN shows the most pronounced changes in FC following sleep onset, and during wakefulness in relation to prior sleep history, we focussed on the functional interactions within the DMN. In addition, we hypothesize that during the different sleep stages FC is likely to be differentially related to objective and subjective measures of habitual sleep.

## 2 METHODS AND MATERIALS

### 2.1 Experimental design

Twenty‐one healthy participants (10 male, 25 ± 3 years) with no reported history of neurological, psychiatric, or sleep disorders completed the study. Participants’ habitual sleep patterns were monitored with a written self‐reported sleep diary and wrist actigraphy (Actiwatch 2, Philips, Respironics®) for two weeks prior to the scanning session. To assess sleep quality, daytime sleepiness, and insomnia symptoms, participants also completed the Pittsburgh Sleep Quality Index (PSQI) (Buysse, Reynolds, Monk, Berman, & Kupfer, 1989), Epworth Sleepiness Scale (ESS) (Johns, 1991), and the Insomnia Severity Index (ISI; Bastien, 2001) questionnaires. Five subjects were excluded for either failing to sleep (three subjects), excessive movement (one subject), or technical problems during electroencephalography (EEG)‐fMRI acquisition (one subject), resulting in a final analyzed cohort of sixteen subjects (eight male, 26 ± 3 years). The study was approved by the University of Birmingham Ethics Committee.

### 2.2 Sleep measures

Actigraphy has been shown to correspond with polysomnographic classification of sleep in the healthy adult population (Ancoli‐Israel et al., 2003; Sadeh, 2011), with a 90% concordance (Sadeh, Hauri, Kripke, & Lavie, 1995), and thus is an accurate and noninvasive way to assess prolonged, habitual sleep habits at home. The raw actigraphy data were firstly compared with the sleep diary for consistency, and two measures of TST (in minutes) were calculated for each subject: two‐week average (ATST) and the TST specifically on the night before scanning (BTST).

Subjective sleep questionnaires were scored according to each validated procedure (PSQI, ESS, and ISI), with higher scores indicating more sleep difficulties or presence of daytime sleepiness. All measures were standardized (*z*‐scored). See Table 1 for the sleep measure demographics. All measures were correlated to investigate the association between each of the objective and subjective sleep measures.

**Table 1:** Demographic information of objective and subjective sleep measures (*N* = 16)

Sleep measures	Mean	Median	*SE*	*SD*	Range
Objective: Actigraphy (mins)					
ATST	397.1	402.5	±10.1	±40.4	316–457
BTST	354.1	358	±23.0	±92.1	120–481
Subjective: Questionnaires					
PSQI	4.6	4.5	±0.5	±1.9	1–7
Epworth	6.4	6.0	±1.0	±3.9	0–14
ISI	4.3	4.0	±0.7	±2.7	0–9

ATST: Average total sleep time; BTST: Before total sleep time; PSQI: Pittsburgh Sleep Quality Index; ISI: Insomnia Severity Index.

### 2.3 EEG‐fMRI data

Neuroimaging has become an increasingly important objective tool for studying the brain during sleep itself (Dang‐Vu, 2012; Duyn, 2012), with the combined recording of EEG and fMRI (EEG‐fMRI) particularly useful for linking standard EEG‐based sleep staging (AASM, 2007) with spatially localized changes in brain function and FC (Wilson et al., 2015). EEG‐fMRI data were simultaneously acquired. EEG data were recorded from 62 Ag/AgC1 MR‐compatible EEG electrodes (EasyCap), with electrodes placed below the left clavicle or on the participant's back to record the electrocardiogram and an additional electrode placed below the left eye to record the electrooculogram. The reference electrode was positioned at FCz, and the ground electrode was positioned at AFz. EEG data were acquired at a sampling rate of 5 kHz, with hardware filters 0.016–250 Hz, using MR‐compatible BrainAmp MR‐plus EEG amplifiers (Brain Products, Germany). Impedance at all recording electrodes was maintained below 20kΩ. The EEG clock was synchronized to the MR scanner clock to ensure consistent sampling of the gradient artifact (Mandelkow, Halder, Boesiger, & Brandeis, 2006). A 3T Philips Achieva MRI Scanner with a 32‐channel head coil was used. Participants wore earplugs and headphones to minimize acoustic noise, and their head was supported with foam padding to minimize motion artifacts.

Subjects were asked to sleep throughout the scan session and to signal using the internal buzzer if they were unable to do so or when they wished to terminate the session. No sleep deprivation protocol was used, and the scanning session took place at the subject's usual bedtime (between 22:00 and 00:00) to try to promote normal sleep. An initial T1‐weighted anatomical scan (1 mm isotropic resolution) was acquired before whole brain T2*‐weighted fMRI data (1,250 volumes, 3 × 3 × 4 mm voxels, TR = 2,000 ms, 32 slices, TE = 35 ms, flip angle 80°, SENSE factor = 2) were acquired in consecutive scans lasting just over 40 min each. Participants underwent between one and three fMRI scans as determined by their indication to terminate the session.

Respiratory and cardiac fluctuations were recorded using a pneumatic belt and a vectorcardiogram (VCG), both acquired at sampling rate of 500 Hz by the scanner hardware. MR gradient and ballistocardiogram artifacts were removed from the EEG using average artifact subtraction in BrainVision Analyzer 2 (Brain Products, Munich). The VCG R‐peak markers were aligned with the EEG data and used for pulse artifact correction (Mullinger, Morgan, & wtell, 2008). EEG data were then divided into nonoverlapping thirty‐second epochs and manually sleep staged by an experienced electroencephalographer using a standard sleep montage according to the American Association of Sleep Medicine criteria (AASM, 2007).

### 2.4 fMRI preprocessing and FC analysis

fMRI data were preprocessed according to standard methodology to prepare for FC analysis (Fox et al., 2005), using FSL (FMRIB Software Library, http://www.fmrib.ox.ac.uk/fsl, Smith et al. 2004) and MATLAB (MathWorks, USA). Data were motion corrected using MCFLIRT, spatially smoothed (using a 6‐mm Gaussian kernel), and temporally band‐pass filtered (0.009 < Hz<0.08). RETROICOR was used (Glover, Li, & Ress, 2000) to reduce the impact of non‐neuronal physiological noise associated with breathing and cardiac fluctuations, and further potential confound signals (six motion parameters of head rotation and translation, white matter, and cerebrospinal fluid signals) were removed using multiple linear regression.

**Figure 1:**
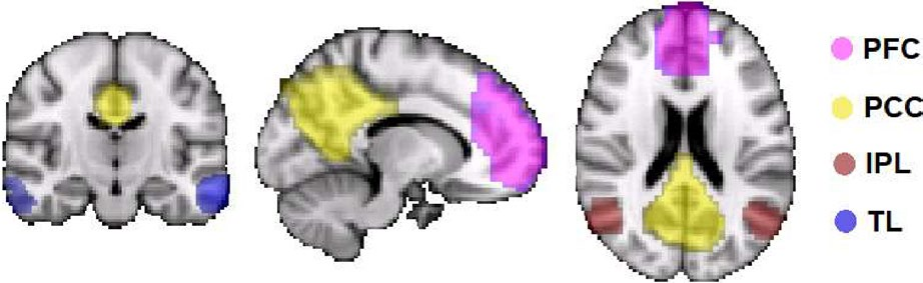
Default mode network regions of interest

Following previous methodology (Khalsa et al., 2016; Khalsa, Mayhew, Chechlacz, Bagary, & Bagshaw, 2014), regions of interest (ROIs) were identified from a group independent component analysis of a separate data cohort to ensure a canonical definition of the key nodes of the DMN (Figure 1). The DMN regions (see Table 2 for MNI coordinates) included the following: posterior cingulate cortex (PCC), mesial prefrontal cortex (mPFC), left inferior parietal lobe (LIPL), right inferior parietal lobe (RIPL), left temporal lobe, and right temporal lobe (RTL). FC analysis was calculated using Pearson correlation and *Z*‐scores calculated between each pair of DMN ROIs using a nonoverlapping 30‐s sliding window of the BOLD time series (Wilson et al., 2015). Within participants, each of these 30‐s FC matrices was paired to the corresponding EEG‐defined sleep stage, pooled by stage, and then averaged across subject epochs.

**Table 2:** Peak voxel coordinates for each regions of interest defined by the group ICA

Network	Region	MNI coordinates		
		*X*	*Y*	*Z*
Default mode network (DMN)	Posterior cingulate (PCC)	2	−54	32
	Prefrontal cortex (mPFC)	2	50	4
	Left inferior parietal lobe (LIPL)	−50	−70	36
	Right inferior parietal lobe (RIPL)	50	−70	36
	Left temporal lobe (LTL)	−62	−12	−20
	Right temporal lobe (RTL)	54	0	−32

ICA: independent component analysis; MNI: Montreal neurological institute.

### 2.5 Statistical analysis

All statistical analyses were conducted on the standardized FC values (*Z*‐scores) using SPSS. Data were normally distributed according to tests of normality (Shapiro–Wilk), as such a repeated‐measures ANOVA assessed FC strength as a factor of regional paired connections (ROI), sleep stage (wake, N1, and N2), and subsequent ROI–stage interactions within the DMN. Finally, a repeated‐measures analysis of covariance with factors ROI, Stage, and ROI*Stage was conducted including each sleep measure (PSQI, ESS, and ISI) as a covariate in the model as a predictor of FC strength. If the covariate was found to significantly interact with the overall FC or any of the factors, subjects were median split into two groups for that sleep measure for descriptive purposes. A false discovery rate was calculated to control for multiple comparisons (Benjamini & Hochberg, 1995). Finally, if the Mauchly's test indicated a violation of the assumption of sphericity within any of the models, the Epsilon Greenhouse–Geisser correction was reported.

## 3 RESULTS

Relationships between the measures of sleep behavior from Table 1 were investigated using Pearson correlation. The only significant positive correlation was between average total sleep time (ATST) and before total sleep time (BTST) *(r* = 0.515, *p* = 0.041), while all other correlations were nonsignificant. This is consistent with the often reported disagreement between objective and subjective assessments of sleep (Baker, Maloney, & Driver, 1999).

We recorded a total of 2,433 thirty‐second epochs of data during EEG‐fMRI, 714 epochs of wake (~5 hr), 1,209 epochs of N1 (~10 hr), 510 epochs of N2 (~4 hr), and 69 epochs of N3 (~30 min). Due to the limited number of N3 epochs recorded in the current dataset and their uneven distribution across subjects, N3 was not analyzed further.

### 3.1 FC changes with sleep stage

An ANOVA found a significant effect of ROI on the FC of the DMN (*F*(14,154) = 22.151, *p* < 0.001, ɳ^2^ = 0.67). Although Stage was not found to be significant (*F*(2,22) = 2.521, *p* = 0.103, ɳ^2^ = 0.19), there was a significant ROI*Stage interaction (*F*(28,308) = 1.921, *p* = 0.004, ɳ^2^ = 0.15) suggesting that changes to DMN FC with sleep stage are regionally specific. Pairwise comparisons revealed a significant FC increase for the PCC‐TL between N1 and N2 (*p* = 0.024), while FC decreased for the mPFC‐lIPL between wake and N1 (*p* = 0.028) and N2 (*p* = 0.043) and for the mPFC‐rIPL between wake and N1 (*p* = 0.018). The bilateral TL significantly increased FC between wake and N2 (*p* = 0.03).

### 3.2 FC association with objective sleep measures

#### 3.2.1 Average total sleep time

The ATST across the 2 weeks prior to the sleep scan was not significantly associated with FC, suggesting that the average nightly sleep duration may not be an influencing factor in FC changes during subsequent sleep.

#### 3.2.2 Night before total sleep time

**Figure 2:**
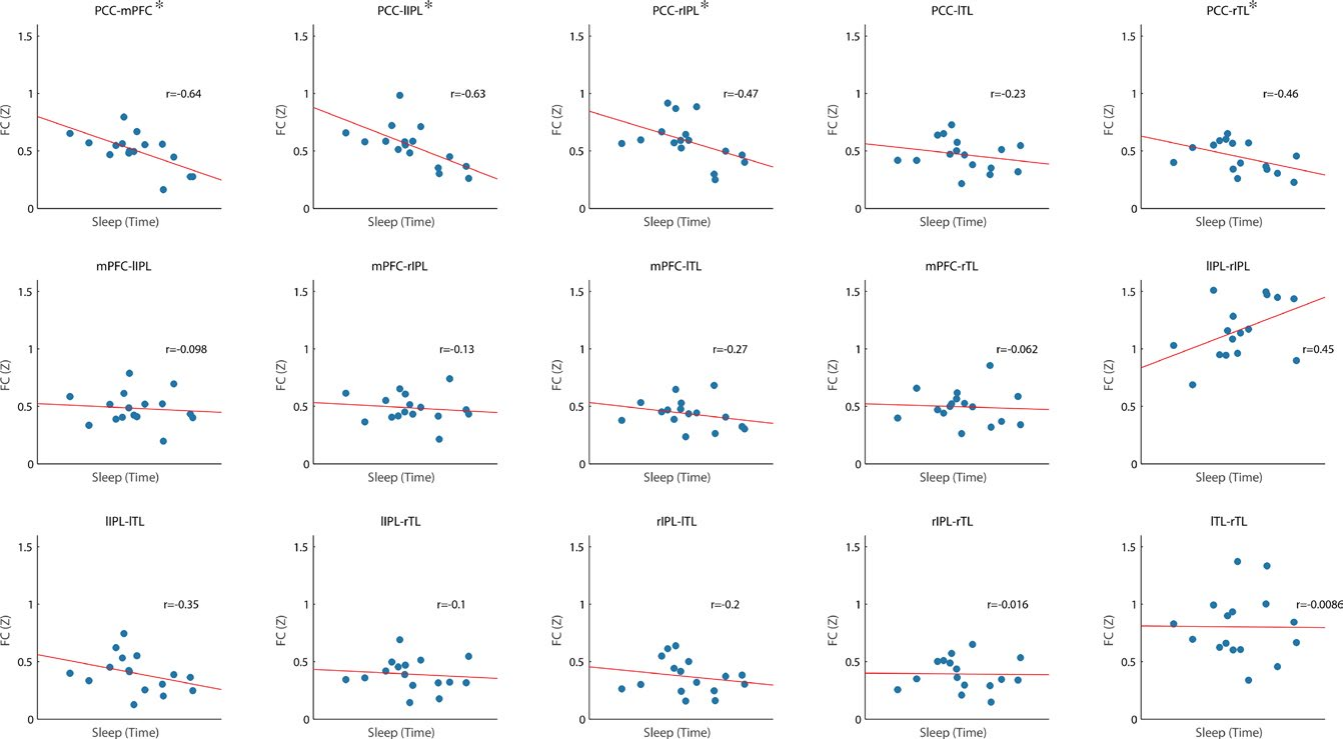
Before total sleep time in minutes and regions of interest interactions upon the strength of functional connectivity within the default mode network, *Significant pairwise connection *p* < 0.05 after false discovery rate

Variability in sleep duration during the night before the sleep scan (BTST) was significantly related to the intersubject FC within the DMN. Before total sleep time significantly interacted only with the main effect of ROI (*p* = 0.002), with pairwise comparisons finding significantly different FC restricted to the PCC, and specifically PCC connections to mPFC, bilateral IPL regions, and RTL (Figure 2). For these regions, less BTST was associated with greater FC, regardless of Stage (i.e., the association between BTST and FC was not impacted by sleep onset or the transition from N1 to N2).

**Figure 3:**
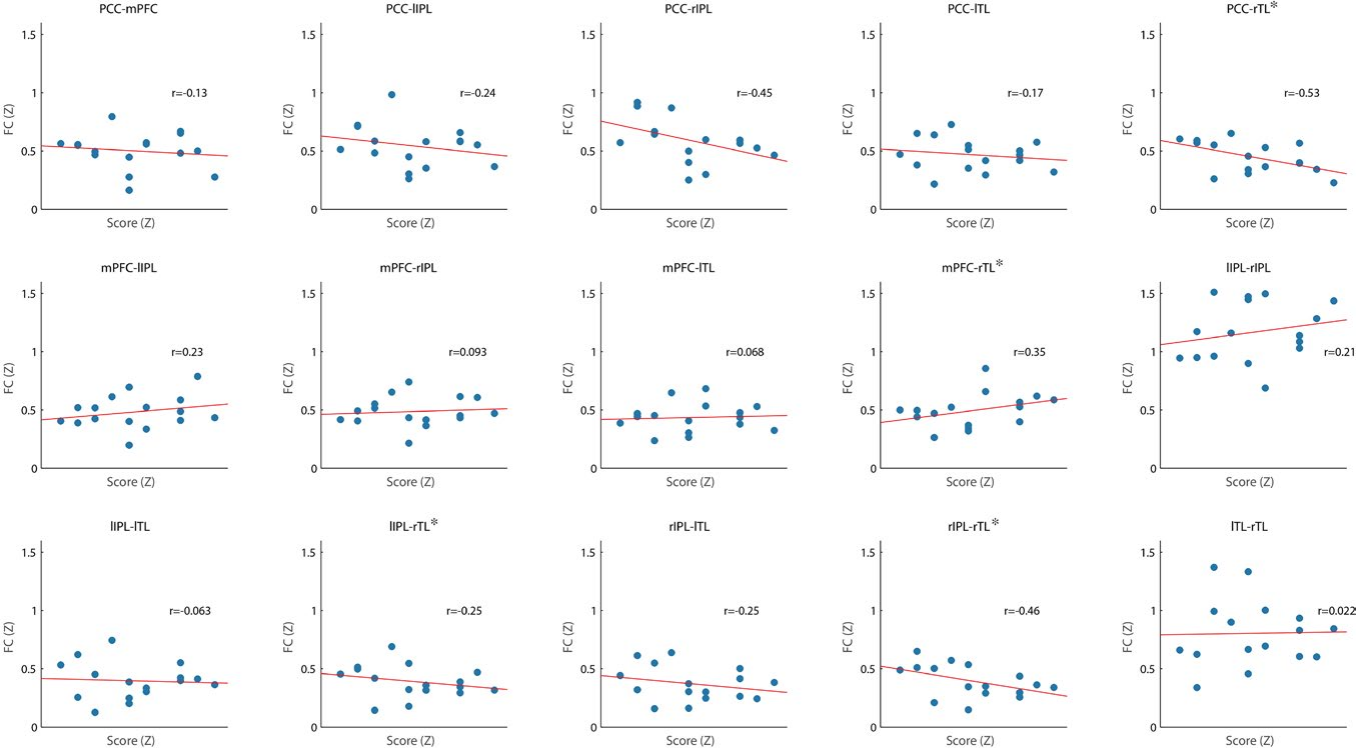
Insomnia Severity Index by regions of interest interaction upon the functional connectivity strength within the default mode network, *Significant pairwise connection *p* < 0.05 after false discovery rate

Neither ESS nor PSQI scores were significantly associated with FC within the DMN suggesting that self‐reported sleep quality and sleepiness were not related to FC changes during sleep. The ISI score was significantly related to FC, as indicated by a significant interaction with ROI (*p* = 0.007), suggesting that the ISI score associated differentially with the FC of the DMN regions. Pairwise comparisons revealed that the RTL FC (to all other DMN regions) was significantly associated with greater ISI scores, see Figure 3.

## 4 DISCUSSION

While FC changes with sleep stage have been well documented (Hale et al., 2016; Horovitz et al., 2009; Larson‐Prior et al., 2009, 2011; Spoormaker et al., 2010; Spoormaker, Czisch, Maquet, & Jancke, 2011), the primary aim of our study was to investigate whether these changes were related to objective and subjective measures of prior sleep. In accordance with these previous reports, we found FC changes with sleep onset and the transition between early non‐rapid eye movement (NREM) sleep stages within the DMN. Furthermore, we have shown that interindividual variability in both objective and subjective measures of prior sleep–wake behavior was associated with FC during subsequent wakefulness and NREM sleep. In addition, just as changes in FC with sleep stage are not homogenous across the brain (e.g., Larson‐Prior et al., 2011), neither are the associations between sleeping FC and prior sleep–wake behavior. Interestingly, in several cases FC was related to sleep measures independently of sleep stage, suggesting FC has contributions from longer term historical sleep behavior that are as pronounced as the more dynamic effects relating to sleep stage. This highlights the advantages of considering both objective and subjective measures of a subject's prior sleep history in studies utilizing FC analysis during wakefulness and sleep, and indicates the complexity of the impact of sleep on the brain both in the short and long term.

### 4.1 Objective sleep measures and DMN FC during sleep

Wrist actigraphy was the main objective measurement used in the current study to assess sleep–wake behavior. The ability of actigraphy to predict FC within the DMN, specifically when seeding in the mPFC, has previously been demonstrated during wake (Khalsa et al., 2016). The current study also found actigraphy measures to be predictive of the FC between regions within the DMN during sleep. Thus, habitual sleep patterns appear to be related to the FC of resting brain networks. To our knowledge, the association between FC during sleep and previous sleep history has not been investigated, and this is the first study to demonstrate a link between what happens in the brain during sleep and previous sleep–wake history. While waking behavior has been shown to impact on the brain during sleep, for example with slow wave activity across cortical areas being linked to the level of prior activity in wake (Huber et al., 2006), similarly our observations highlight that FC during sleep is linked and modulated by prior sleep behaviors.

The total amount of sleep on the night before the scanning session (BTST) was related to greater FC across the majority of DMN regions, with greater FC associated with a shorter sleep duration. This effect was regionally dependent, being specific to FC of the PCC to other DMN regions, and also observed as an interaction between BTST and ROI, rather than with Stage. This suggests that the association between BTST and FC was not impacted by the transitions between wake and sleep, or between sleep stages. The importance of prior sleep for PCC FC is supported by previous research showing reductions in PCC FC with increased sleep pressure from partial sleep deprivation (Sämann et al., 2010), as well as further reductions in DMN FC more generally with sleep deprivation during wake (Gujar et al., 2010; Yeo et al., 2015). We show that this decrease in DMN FC with increased sleep pressure is different when sleep itself is included, with less sleep the night before scanning associated with higher FC. This interpretation in terms of sleep pressure relies upon the assumption that people with shorter sleep duration have a sleep debt and hence increased sleep pressure, a link which has been observed and suggested to be the result of variation in sleep duration between individuals driven by self‐selected sleep restriction (Klerman & Dijk, 2005). Interestingly, both time of day and sleep deprivation have been shown to impact waking FC within the DMN, with increased FC strength in wake following sleep deprivation (Kaufmann et al., 2015). In addition, FC changes in the DMN in people with insomnia were found to be significantly negatively correlated with sleep efficiency, that is, individuals with poor sleep efficiency were more likely to have greater FC in the DMN during wake (Regen et al., 2016).

The current study is the first to suggest that FC during sleep is also influenced by prior sleep habits, and, assuming the link between sleep duration and sleep debt, it would suggest that increased sleep pressure (i.e., shorter sleep duration) increases DMN FC during subsequent sleep. However, the lack of a significant effect of sleep stage on the BTST makes this a speculation which must be clarified in future studies, with further research needed to directly compare FC between daytime wake and during sleep as a function of sleep debt, to identify a potential *switch* in FC strength. Interestingly, ATST was not predictive of FC during sleep for the DMN, suggesting that either it is not a sensitive measure of variability in sleep history or only recent sleep (i.e., BTST) impacts on DMN FC.

### 4.2 Subjective measures and the DMN FC during sleep

There are several ways of quantifying sleep subjectively, and the current study used three of the most common ones for clinical applications: the PSQI (Buysse et al., 1989), ESS (Johns, 1991), and the ISI (Bastien, 2001). Of these, only the ISI was found to be predictive of differences in DMN FC, specifically FC of the RTL to all regions within the DMN was reduced in subjects reporting a greater level of insomnia symptomatology. Although none of our participants explicitly experienced insomnia itself, our observation is consistent with the theory that DMN FC could be linked with insomnia more than other brain networks (Marques, Gomes, Caetano, & Castelo‐Branco, 2018). It has also been suggested that the subjective experience of insomnia is associated with overactivation within the DMN, particularly the PCC, during NREM sleep (Buysse, Germain, Hall, Monk, & Nofzinger, 2011). The association between ISI scores and TL FC, a region involved in memory function (Alvarez & Squire, 1994), could underlie the impairments in memory performance noted in people with insomnia (Backhaus et al., 2006; Nissen et al., 2006, 2011). Thus, the finding that reduced FC of the TL during sleep is associated with greater reported symptoms of insomnia requires further investigation.

It is important to note that none of the participants exceeded the diagnostic criteria for insomnia, and as such, the statistically significant result in our study cannot be interpreted as clinically meaningful. However, insomnia symptoms are highly prevalent in the nonclinical population (Ohayon, 2002; Ohayon & Reynolds, 2009) and are associated with daytime impairments including in memory (Ohayon, 2009). A recent study focusing on waking DMN FC in individuals with insomnia did not find any significant differences compared with healthy controls or an association with subjective measures of sleep efficiency (Regen et al., 2016). Our observations may suggest that FC differences in insomnia could be more pronounced during sleep rather than in wake. While a different pathology, the idea that the change in state from wakefulness to sleep can uncover FC differences between patient groups and healthy participants is supported by our recent work in idiopathic generalized epilepsy (Bagshaw et al., 2017). Additionally, the current study was limited in not being able to assess slow wave sleep, which is understood to be both important for memory consolidation and is influenced by insomnia (Diekelmann & Born, 2010). Therefore, the relationship with clinical insomnia and the assessment of DMN FC in SWS are both interesting areas for future investigation.

### 4.3 Methodological considerations and future research

The current study is limited to the first few cycles of sleep, and FC could differ in the relationship to prior sleep measures in later cycles. Indeed, fMRI differences have been noted across N1 cycles (Picchioni et al., 2008). Therefore, future work to assess the variability in FC within an EEG‐classified sleep stage is an important question which can be assessed using the dynamic FC analysis that we have employed. In addition, extending scanning to later in the night or continually across the whole night would enable an even greater idea of the FC changes occurring during sleep and their relationship with prior sleep behavior, although this presents a challenge within the scanner environment.

## 5 CONCLUSION

FC of the DMN is associated with objective and subjective measures of prior sleep history in a regionally specific manner. While sleep onset is associated with changes to FC of the DMN, differences in FC are also predicted by the characteristics of an individual's prior sleep. This suggests independent contributions to variability in FC from dynamic, state‐dependent processes as well as more stable, trait‐like features. This finding has implications for our understanding of the cumulative effects of the sleep–wake cycle on brain function, suggesting that FC may reflect more cumulative or historical information about the sleep–wake cycle than previously thought. This may influence FC studies focusing on clinical populations known to experience cumulative sleep deprivation or disruption (e.g., psychiatric disorders or neurogenerative disease) (Wulff, Gatti, Wettstein, & Foster, 2010). In particular, further research into sleep FC with those diagnosed with insomnia could provide valuable insights into the mechanism of cognitive and behavioral changes associated with the condition.
